# An in vivo Insect-based Platform for Preclinical Endoscopic Imaging

**DOI:** 10.1055/a-2967-5493

**Published:** 2026-10-07

**Authors:** Ronaldo A. González García, Adrian P. Regensburger, Benno Weigman, Sophie Wilhelm, Maximilian J. Waldner, Joachim Wölfle, Gabriele A. Krombach, Ferdinand Knieling, Anton G. Windfelder, Hannah Vogt-Wolz

**Affiliations:** 1 Pediatrics and Adolescent Medicine, Friedrich-Alexander-Universität Erlangen-Nürnberg (FAU) 27168 Universitätsklinikum ErlangenErlangenBYGermany; 2 Medicine 1, Friedrich-Alexander-Universität Erlangen-Nürnberg (FAU) 27168 Universitätsklinikum ErlangenErlangenBYGermany; 3 Department of Diagnostic and Interventional Radiology (Experimental Radiology) 14973 University Hospital GiessenGiessenGermany; 4 Department of Radiology 420479 Biruni UniversityIstanbulTurkey; 5 Department of Bioresources 652666 Fraunhofer Institute for Molecular Biology and Applied Ecology IME Branch for BioresourcesGiessenHEGermany

**Keywords:** endoscopy small bowel, inflammatory bowel disease, endoscopy lower GI tract

## Abstract

**Background and Study Aim** Advanced endoscopic imaging technologies are routinely optimized and validated using vertebrate animal models. We present a novel, high-throughput insect model that provides a biologically realistic, reproducible, and ethically unproblematic alternative for evaluating endoscopic imaging modalities.

**Methods***Manduca sexta* larvae (fifth instar, 6.6 ± 0.23 g) were used for the demonstration of endoscopic imaging. Healthy controls ( *n* = 27) were compared with chemically (5% dextran sodium sulfate, *n* = 20) and bacterially ( *Bacillus thuringiensis* , *n* = 33) induced gut inflammation.

**Results** Small-animal endoscopic imaging was readily feasible and revealed distinct anatomical features of *M. sexta* larvae. Animals with chemical-induced inflammation exhibited rectal swelling, melanization throughout the intestine, and increased transparency of the midgut epithelium. In comparison, bacterially induced inflammation resulted in intestinal swelling, a less extensive transparent membrane, melanization, and lesions in the hindgut and posterior midgut.

**Conclusion** Endoscopic imaging of the intestinal tract of *M. sexta* larvae enabled a comprehensive examination of the internal anatomy under different inflammatory conditions. *M. sexta* larvae provide a suitable in vivo platform for studying intestinal inflammation over time. The model offers high survival rates, strong reproducibility, and cost-effectiveness, supporting the reduction of vertebrate animal experiments in accordance with the Replacement, Reduction, and Refinement principles.

## Introduction

Advances in endoscopic imaging technologies have enabled increasingly precise visualization of mucosal structures and molecular processes in vivo. Techniques such as confocal laser endomicroscopy, hyperspectral and fluorescence endoscopy, and molecular probe–based imaging now enable real-time assessment of cellular and subcellular alterations during disease progression. 1 2 3 4 However, the translation of these methods from the laboratory to clinical application remains dependent on suitable preclinical models that accurately replicate biological complexity while complying with ethical and practical constraints.

Traditional vertebrate animal models, preferentially murine models, pose challenges related to animal welfare, cost, and experimental throughput. In recent years, alternative invertebrate systems have emerged as promising platforms for translational research, supporting the principles of the Replacement, Reduction, and Refinement (3Rs). Among these, the *Manduca sexta* (tobacco hornworm) larva has been introduced as a novel model for inflammatory bowel disease, 5 6 exhibiting conserved disease immune and signaling pathways with up to 92% genetic homology to humans. 7 The larva’s substantial size and comparable gut volume to that of the mouse provide unique opportunities for endoscopic access and imaging-based investigation. 8 9

Previous studies employing *M. sexta* have primarily used radiological techniques, such as computed tomography (CT) and magnetic resonance imaging (MRI), to characterize structural changes, including gut wall thickening after inflammatory induction. 5 Yet, direct endoscopic visualization of the intestinal mucosa and its dynamic response to inflammation has not been achieved. Establishing endoscopic imaging in this model would enable high-resolution, potentially molecular-level visualization of inflammatory processes, complementing existing virtual datasets and allowing longitudinal monitoring with minimal ethical burden, cost, and therefore high sustainability.

The present study therefore aims to establish a novel endoscopic imaging approach for *M. sexta* larvae as a biologically realistic and ethically compliant platform for preclinical evaluation of advanced and molecular imaging techniques.

## Materials and Methods

In contrast to the approval requirements for vertebrate research in Europe and the United States, no such approval is necessary for working with insects. *M. sexta* larvae in the fifth instar (developmental days 5 to 6, L5d5/6, 6.6 ± 0.23 g) were investigated endoscopically. The insects were reared with a 16 h photoperiod at 24 °C and fed an additive-free modified artificial diet 5 ( Fig. 1 ). Healthy controls ( *n* = 27), either in the wandering phase, when they generally stop feeding, or as L5d5/6 larvae, were given a regular diet of food cuboids. To induce bacterially or chemically induced 10 gut inflammation, the food cuboids were either submerged in a 0.3% (w/v) *Bacillus thuringiensis* subsp. *aizawai* (Bt) suspension (2.7 × 10 ^8^ cells/mL) (Xentari, Neudorff, #00592) ( *n* = 33), or 5% (w/v) dextran sodium sulfate (DSS; MP Biomedicals, #9030143409) was added to the regular diet ( *n* = 20).

**Fig. 1:**
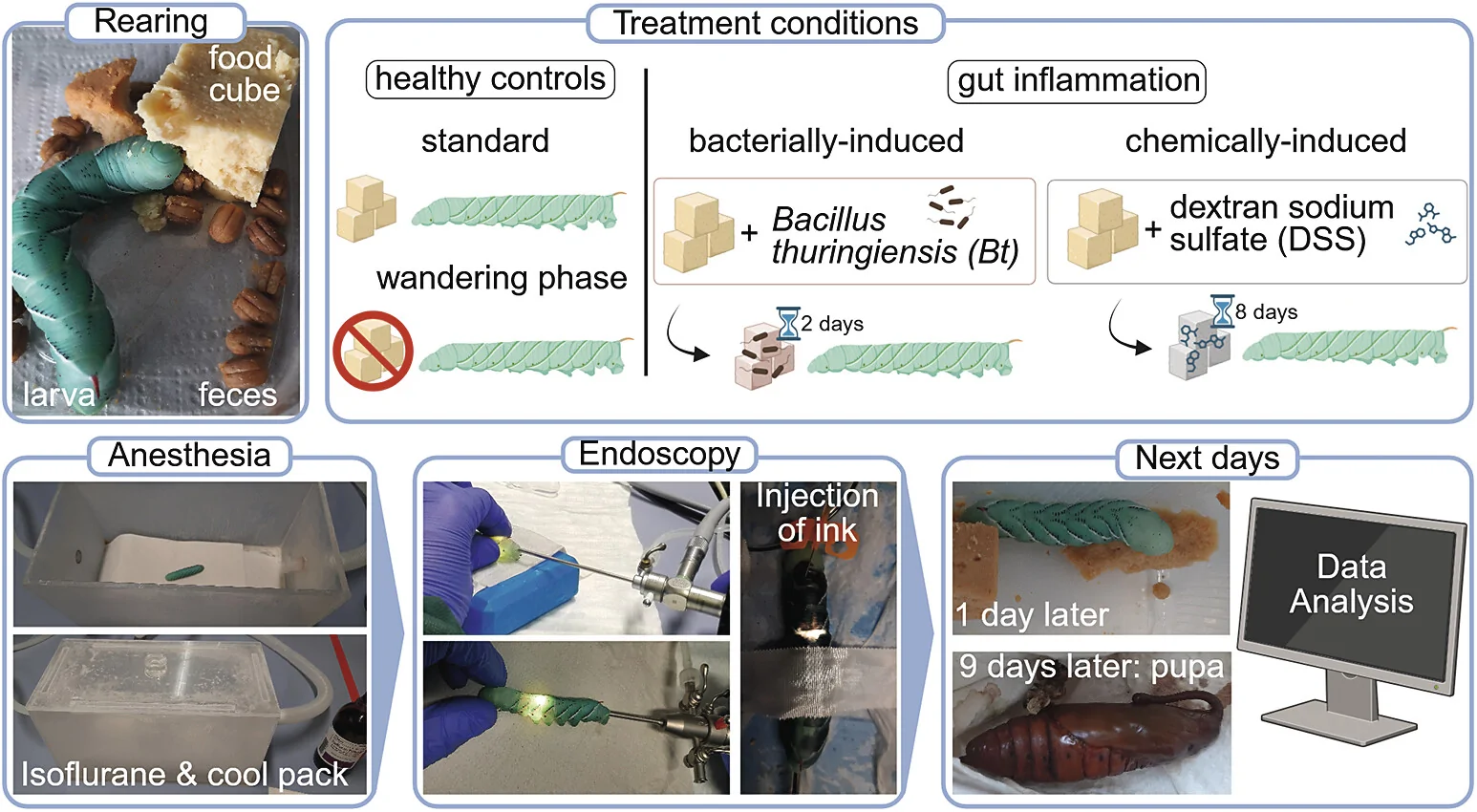
Larval rearing and endoscopy methodology. Rearing: The *Manduca sexta* larvae are reared on an artificial insect diet. The fecal pellets produced by the larvae are hexagonal and adopt the structural geometry of the ileum and colon. Treatment conditions: Four conditions were examined: healthy controls, including standard conditions and the wandering phase, as well as two distinct gut inflammation conditions, including the incorporation of *B. thuringiensis* (Bt) for 48 h to induce bacterial inflammation, and the incorporation of dextran sodium sulfate (DSS) for 8 days to induce chemical inflammation. Anesthesia: The larvae were anesthetized using a combination of isoflurane and a cool pack. Endoscopy: The endoscopy was performed using an adapted mouse endoscope system. Indian ink was injected intravenously during endoscopy for contrast. Next days: The larvae were monitored for normal development until pupation stage and endoscopic imaging data were analyzed. Created in BioRender. Vogt, H. (2026) https://BioRender.com/0s0k2u1 [rerif]

Bt-infected larvae were endoscopically imaged 48 h postinfection, and DSS-exposed larvae were imaged 8 days postfeeding. On the day of the endoscopy, the caterpillars were weighed (healthy: 8.38 ± 0.34 g; Bt: 5.95 ± 0.22 g; DSS: 5.21 ± 0.40 g) and then placed into a chamber on top of a cool pack and cloth. In addition to cooling, the insects were anaesthetized by applying approximately 1 mL of isoflurane to the cloth positioned adjacent to the animal. After a brief period, the animals were prepared for endoscopy. Fecal pellets were manually extracted from the rectum. A standard mouse setup (COLOVIEW Miniendoscope System Mainz, Karl Storz, Tuttlingen, Germany), as previously described by Becker et al., 11 12 was utilized and adapted for larval examination. All endoscopic examinations were performed using conventional white-light illumination provided by the COLOVIEW Miniendoscope System. No optical image-enhancement modalities, including narrow-band imaging, chromoendoscopy, fluorescence imaging, or digital postprocessing enhancement, were applied during image acquisition. Briefly, a 1.9-mm endoscopic sheath, 10 cm in length, was attached to a 0° straightforward telescope (HOPKINS, Karl Storz) connected to a camera head. Illumination was provided by a cold light fountain (POWER LED 175 SCB) set to 100% capacity. Prior to imaging, the air pump flow rate was individually calibrated in a water bath to produce approximately one bubble per second via adjustment at the sheath adapter. During the procedure, larvae were positioned on a cool pack. The endoscope was inserted rectally while gently inflating the hindgut and midgut. Upon reaching the anterior midgut, airflow was turned off, and the endoscope was slowly withdrawn to capture high-resolution video footage of the entire gastrointestinal tract. Videographic documentation was obtained during the procedure. The assessment of epithelial integrity was performed by injecting black ink into the dorsal vessel (main, open vessel of insects, also known as the heart) of larvae in the wandering phase during endoscopy.

The endoscopic videos were analyzed using Adobe Premiere Pro 2024, a video-editing software. This analysis involved extracting images from the videos.

## Results

The generation time of *M. sexta* is relatively short. From hatching until the pupa stage, it takes around 20 days, with five instar stages in between, each roughly 2–5 days. 13 The fifth instar is the final stage before pupation, when the larva is at its largest. This stage was therefore chosen for testing the accessibility of endoscopy. The dimension of the larva on the last day of the fifth instar (L5d6) is shown in Table 1 . In the transition state from the fifth instar stage to pupa, the larvae are entering the wandering phase, a developmental stage characterized by cessation of feeding, gut purging, and the search for a protected site for pupation. During pupation, the animal gets smaller, and its gut also changes in preparation for the metamorphosis. 14

**Table 1:** Dimensions of *Manduca sexta* larva in the fifth instar on day 6.

LD5d6 larva	Whole body	Hindgut	Midgut
Mean area (mm ^2^ )	4955 ± 1279 ( *n* = 10) 9	348.2 ± 105.1 ( *n* = 10) 9	1289 ± 369.9 ( *n* = 10) 9
Mean volume (mL)	6.546 ± 2.556 ( *n* = 10) 9	0.208 ± 0.086 9	1.40 ± 0.465 9
Mean length (cm)	7.5-9.0 ( *n* > 40) 13		
Mean mass (g)	8.25 ± 1.5 ( *n* > 40) 13		

To confirm the feasibility of endoscopic imaging of the gut in *M. sexta* larvae, fifth-instar individuals in the wandering phase were examined first due to their naturally empty gut. As expected in this stage, the midgut is free of food remnants and lacking a peritrophic matrix (a transient, mucus-like luminal barrier containing structural chitin). Consequently, the gut epithelium was directly accessible for inspection, and virtual digestive tract segmentation was used to orient in the specimen ( Fig. 2A ). 9 The midgut epithelium appeared as a white, transparent membrane with delicate folds or plicae ( Fig. 2B ). Despite having fasted for a period of 2 h before undergoing an endoscopy, L5d5/6 larvae (not in the wandering phase) exhibited food remnants. Although video acquisition was frequently feasible, recordings were limited to the upper portion of the midgut lining, comprising the midgut epithelium and the peritrophic matrix. For clarity, the midgut epithelium and the peritrophic matrix are hereafter collectively referred to as the “gut membrane.”

**Fig. 2:**
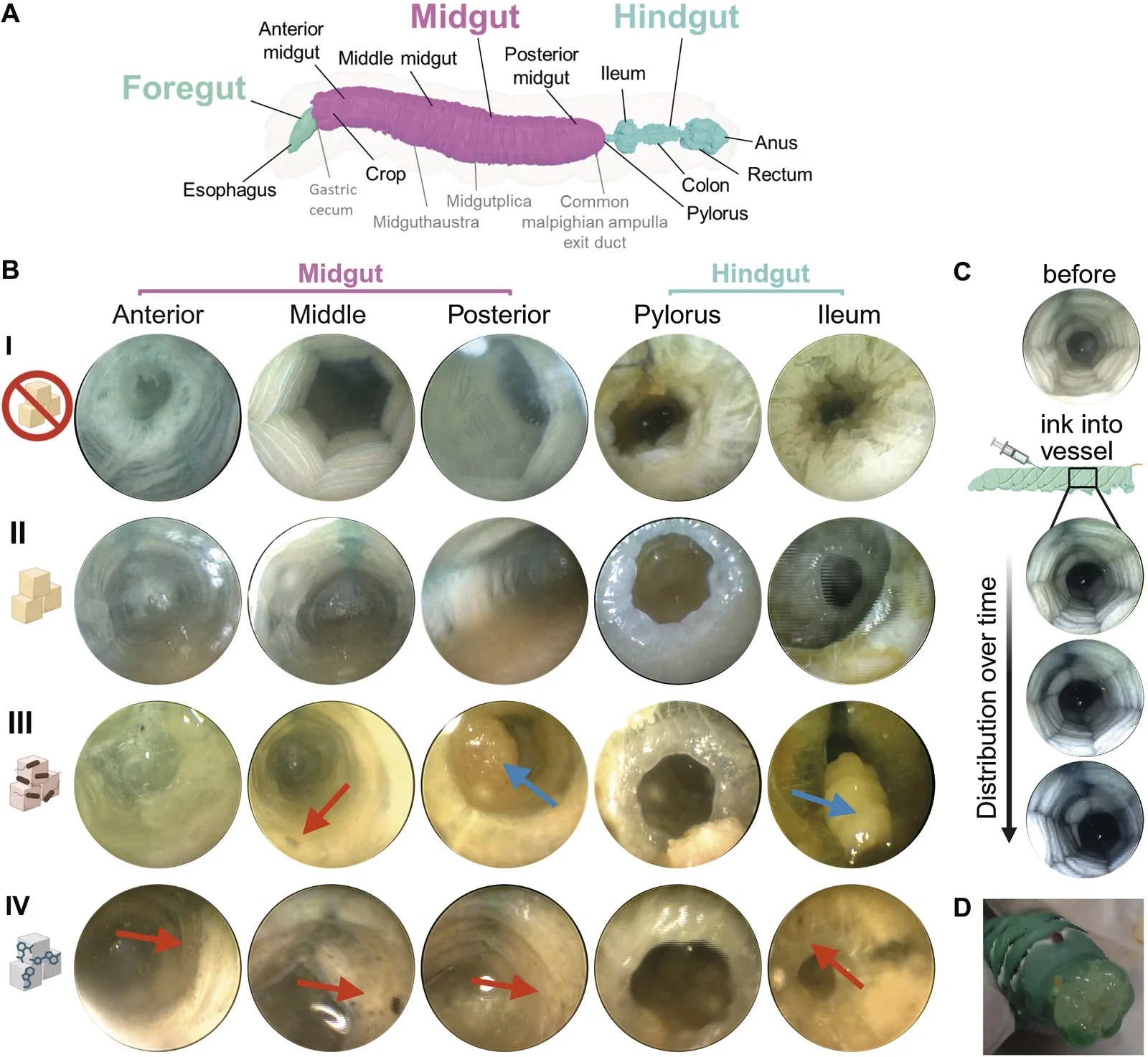
Correlation of results of larval endoscopy with intestinal structure and various inflammatory conditions. ( **A** ) The virtual digestive tract segmentation based on the μCT data reproduced from Windfelder et al., iScience 2023, under the terms of the Creative Commons Attribution 4.0 International License (CC BY 4.0). 9 ( **B** ) The following representative endoscopic images are provided: (I) healthy larva in the wandering phase (II) healthy larva, (III) bacterially or (IV) chemically induced gut inflammation. The red arrows denote melanization, indicated by black dots, while the blue arrows indicate lesions. ( **C** ) In the experiment, Indian ink was injected into the dorsal (main) vessel of a wandering larva. This method demonstrated that the ink is distributed evenly around the midgut without leaking into the gut over time. ( **D** ) The larva has developed a prolapse that does not hinder the feasibility of the model system. Created in BioRender. Vogt, H. (2026) https://BioRender.com/v7ij607 [rerif]

The light yellowish-green, slightly transparent lining of the hindgut enabled visualization of the organs located above the intestine. The three sphincter muscles appeared white and opened automatically when airflow was applied. The midgut was semitransparent and light brown in color, allowing visibility of the darker, pigmented dorsal vessel (heart) above it and the white fat body (functionally analogous to adipose tissue and the liver), as well as the Malpighian tubules (functionally analogous to kidneys) and trachea (the insect respiratory system) surrounding it. The epithelial structure exhibited a less pronounced division into hexagonal segments compared with the wandering phase, yet the midgut haustrum and plicae remained distinctly discernible. 9

Larvae that were infected with Bt for 48 h exhibited a swollen intestinal region. The lining of the hindgut manifested as robust and yellowish, while the underlying organs became more distinguishable and exhibited a whitish-yellowish hue through the semitransparent membrane. Lesions in the form of gut membrane erosion were also visible. The midgut exhibited a loss of transparency, acquiring a stronger yellowish-brown hue, accompanied by sporadic black dots (melanization, an innate immune reaction characterized by localized pigment deposition 5 ). In certain instances, small amounts of food persisted in the midgut, and the lesions frequently extended from the hindgut to the posterior midgut.

Larvae administered 5% DSS for 8 days exhibited rectal swelling, which made endoscopic access to the gut more time-consuming. The hindgut lining exhibited a yellowish hue and reduced transparency in comparison to that observed in healthy larvae. The gut membrane was more transparent, with minimal or no food remnants, resembling the wandering phase. The presence of dark spots (melanizations, see above) was observed on the midgut epithelium and, on a less frequent basis, in the hindgut.

The injection of black ink into the dorsal vessel of healthy larvae in the wandering phase resulted in the ink being evenly distributed around the gut with no leakage ( Fig. 2C ).

Prolonged irritation of the hindgut, whether through manual cleaning or pipetting water, can result in prolapse and, in some cases, the excretion of the peritrophic matrix ( Fig. 2D ). The latter offers the possibility of directly visualizing the midgut epithelium.

Furthermore, the experiment included six larvae, which underwent a series of endoscopies to assess their resilience. For this purpose, the subjects underwent endoscopy on two consecutive days. The caterpillars exhibited excellent survival rates (100%) following both procedures. In general, the larvae survived endoscopy without complications, except when the peritrophic matrix has detached and is expelled (which occurred twice during the study) or the intestine has been accidentally perforated. Even a prolapse was usually survived by the caterpillars.

## Discussion

This study demonstrates the feasibility of endoscopic imaging in *M. sexta* larvae, providing direct visualization of the larval intestinal architecture and its inflammatory alterations. The ability to perform minimally invasive, repeated imaging within the same organism establishes this model as a valuable platform for longitudinal studies, aligning with the principles of the 3Rs.

Endoscopic visualization revealed a well-defined compartmentalization of the digestive tract, distinguishing the hindgut, including the rectum, colon, and ileum, as well as the pylorus and the hexagonal microstructure of the posterior, middle, and anterior midgut. These findings align closely with prior virtual segmentation of the digestive tract from CT and MRI data, confirming anatomical consistency across imaging modalities. 9 14 This highlights the potential of *M. sexta* for multimodal validation of emerging endoscopic and molecular imaging technologies. 1 2 3 4 15 In the future, this model may enable high-throughput evaluation of molecular imaging approaches. Physiological variation among larval stages influenced luminal visibility, with food remnants decreasing markedly in the wandering phase. This stage-dependent clearance of intestinal contents enables superior endoscopic assessment and should be standardized in future imaging protocols. Importantly, endoscopy did not interfere with larval development or survival, permitting repeated imaging at multiple time points, an essential feature for investigating dynamic inflammatory processes or therapeutic interventions. The two inflammation models, chemically and bacterially induced, produced distinct endoscopic phenotypes. Chemical induction primarily resulted in rectal swelling and transparent gut membranes with scattered black spots indicative of melanization. 5 16 In contrast, bacterially induced inflammation resulted in erosive changes, membrane thickening, and reduced transparency, predominantly affecting the midgut. These differences likely reflect distinct immune pathways and barrier responses, consistent with known insect innate immune mechanisms, such as melanization 5 16 and epithelial remodeling. 17 Importantly, these macroscopic endoscopic findings closely resemble the principles of visual inflammatory assessment routinely performed during clinical gastrointestinal endoscopy, where changes in tissue architecture, edema, and mucosal integrity serve as indicators of disease activity. In the *M. sexta* model, reduced transparency most likely reflects inflammatory edema and epithelial remodeling, whereas melanization represents a localized innate immune response to tissue injury. Although melanization is insect-specific, it provides a reproducible optical marker of intestinal inflammation. Consequently, these characteristic imaging features offer objective imaging endpoints that can be used to evaluate novel endoscopic imaging technologies, image-enhancement methods, fluorescence probes, and molecular imaging approaches before their validation in vertebrate models. These findings establish a reproducible baseline against which future imaging-enhancement and molecular imaging techniques can be objectively compared.

From a translational perspective, these findings emphasize the value of *M. sexta* as a biologically relevant and ethically sustainable complement to vertebrate models in the preclinical evaluation of advanced and molecular endoscopic imaging systems. The optical accessibility, structural similarity to vertebrate gut organization, and capacity for longitudinal observation make this model particularly suitable for validating novel contrast agents, fluorescence probes, and label-free molecular imaging modalities. 6 18 19 20

This model serves as a high-throughput preclinical prescreen prior to mouse models. Its rapid reproductive cycle and the ability to endoscopically examine a substantial number of animals within a single day enable rapid screening. Specifically for molecular imaging, the model enables efficient validation of novel endoscopic contrast agents and imaging modalities with minimal effort. Researchers can endoscopically monitor the innate immune response and track therapeutic interventions in real time. For instance, the recruitment of insect immune cells (hemocytes) or the overexpression of luminal proteins (e.g., Duox) 5 during inflammation can be visualized directly. Consequently, this model efficiently refines parameters before validation in higher-order mammalian models. In conclusion, this study establishes the *M. sexta* larva as a viable in vivo platform for endoscopic and potentially molecular imaging of intestinal inflammation. By combining biological relevance with ethical and logistical advantages, this model offers a promising avenue for refining early-stage testing and development of innovative endoscopic imaging technologies.

## Declaration of GenAI Use

The authors confirm that no Generative AI was used in the preparation of this manuscript.
